# Synthesis of K-Carrageenan Flame-Retardant Microspheres and Its Application for Waterborne Epoxy Resin with Functionalized Graphene

**DOI:** 10.3390/polym11101708

**Published:** 2019-10-17

**Authors:** Na Wang, Haiwei Teng, Fei Yang, Jiaqi You, Jing Zhang, Deyi Wang

**Affiliations:** 1 Sino-Spanish Advanced Materials Institute, Shenyang University of Chemical Technology, Shenyang 110142, China; 18842411721@163.com (H.T.); yangfei930212@sina.com (F.Y.); iamyoujiaqi@163.com (J.Y.); zhangjingcszx@syuct.edu.cn (J.Z.); 2 Advanced Manufacturing Institute of Polymer Industry (AMIPI), Shenyang University of Chemical Technology, Shenyang 110142, China; 3 IMDEA Materials Institute, C/Eric Kandel 2, 28906 Getafe, Madrid, Spain

**Keywords:** k-carrageenan, microsphere, waterborne epoxy, flame retardancy

## Abstract

In this article, the intumescent flame-retardant microsphere (KC-IFR) was prepared by inverse emulsion polymerizations, with the use of k-carrageenan (KC) as carbon source, ammonium polyphosphate (APP) as acid source, and melamine (MEL) as gas source. Meanwhile, benzoic acid functionalized graphene (BFG) was synthetized as a synergist. A “four-source flame-retardant system” (KC-IFR/BFG) was constructed with KC-IFR and BFG. KC-IFR/BFG was blended with waterborne epoxy resin (EP) to prepare flame-retardant coatings. The effects of different ratios of KC-IFR and BFG on the flame-retardant properties of EP were investigated. The results showed that the limiting oxygen index (LOI) values increased from 19.7% for the waterborne epoxy resin to 28.7% for the EP1 with 20 wt% KC-IFR. The addition of BFG further improved the LOI values of the composites. The LOI value reached 29.8% for the EP5 sample with 18 wt% KC-IFR and 2 wt% BFG and meanwhile, UL-94 test reached the V-0 level. In addition, the peak heat release (pHRR) and smoke release rate (SPR) of EP5 decreased by 63.5% and 65.4% comparing with EP0, respectively. This indicated the good flame-retardant and smoke suppression property of EP composites coating.

## 1. Introduction

Flame-retardant coatings can effectively reduce the flammability of the coated substrate, prevent the rapid spread of fire, or increase the fire endurance of the coated substrate [1,2,3]. Among them, the intumescent flame-retardant coating consists of a polymer matrix, an intumescent flame-retardant (IFR) system, and a synergist. Due to its high flame-retardant efficiency, it has become an important variety in flame-retardant coatings. The traditional IFR system consists of the mixture of pentaerythritol (PER), ammonium polyphosphate (APP), and melamine (MEL) [4,5,6]. However, the traditional IFR has some unavoidable problems, for example, APP has poor compatibility with polymer matrix and is easily migrated. Microencapsulation of APP can effectively improve the compatibility between APP and polymers. In our previous research, double-shell co-microencapsulated APP and expandable graphite were prepared by the in situ polymerization process with the use of melamine–formaldehyde resin and organic silicon, which improved the compatibility of APP with rubber, thermal stability, and flame retardancy of rubber [7,8].

The proper addition of synergist can effectively enhance flame-retardant efficiency of IFR. Graphene oxide (GO) can be used as a synergistic agent to improve flame-retardant efficiency and reduce smoke emission [9,10,11]. It has been reported that the use of a small amount of GO as a synergist to prepare PBS composites showed a significantly increased limiting oxygen index (LOI) value [12]. However, due to the easily agglomeration GO within the polymer, the flame-retardant efficiency was still highly limited. In recent years, in order to improve the dispersion of GO, many works related to functionalization of graphene oxide have been explored, which showed good interfacial compatibility with epoxy resins and thus provide enhanced flame-retardant efficiency for the IFR system [13,14,15].

In recent years, with the lack of petroleum resources and the enhancement of people’s awareness of environmental protection, biomass carbon sources have gradually emerged [16,17,18]. Therefore, we reported the preparation of flame-retardant microspheres (K-CM/APP) by covering APP with the k-carrageenan (KC) wall. The effect of as synthesized K-CM/APP on the flame retardancy of waterborne epoxy resins was studied [19]. However, the low char layer expansion and poor char layer quality still restricted its property. Therefore, in this work, the addition of MEL was further applied to overcome this restriction. Using MEL as the gas source, KC as the carbon source, APP as the acid source, and benzoic acid functionalized graphene (BFG) as a synergist in waterborne epoxy resin at the same time to prepare the high efficient flame-retardant coating. By Fourier transform infrared spectroscopy (FTIR), scanning electron microscopy (SEM), and X-ray powder diffraction (XRD), the structures of KC-IFR and BFG were characterized. The effects of KC-IFR and BFG and its ratio on flame retardancy, smoke suppression performance, and thermal decomposition behavior of EP were studied systematically with different methods.

## 2. Materials and Methods

### 2.1. Materials

KC was made according to the literature [20]. K-CM/APP was made according to the literature [19]. Graphite oxide and sodium borohydride were obtained from Zhejiang Jiaxing Maya Reagent Co., Ltd. (Jiaxing, China). Anhydrous ethanol, concentrated sulfuric acid, and hydrochloric acid were obtained from Tianjin Yongda Chemical Reagent Co., Ltd. (Tianjin, China). Hydrogen peroxide, potassium permanganate, and sodium dodecyl benzene sulfonate (SDBS) were obtained from Liaoning Jiacheng Fine Chemicals Co., Ltd. (Shenyang, China). Sodium hydroxide, sodium nitrite, and para-aminobenzoic acid were obtained from Tianjin Oubokai Chemical Co., Ltd. (Tianjin, China). Epichlorohydrin and chloroform were obtained from Tianjin Damao Chemical Reagent Factory (Tianjin, China). Span 80 was obtained from Tianjin Ruijinte Chemical Co., Ltd. (Tianjin, China). Cyclohexane was obtained from Tianjin Fuyu Fine Chemical Co., Ltd. (Tianjin, China). Resin 6520-WH-53 and curing agent 8545-W-52 were obtained from Hexion Specialty Chemicals, Inc. (Columbus, OH, USA).

### 2.2. Methods

#### 2.2.1. Synthesis of KC-IFR

Preparation of monomer aqueous solution: A total of 0.3 g KC was dissolved in 10 mL distilled water in a 95 °C water bath. The pH value of the above solution was then adjusted to 7 with 1% sodium hydroxide solution. Then a total 0.1 g of APP and 0.1 g of MEL were added into the solution and stirred until completely dissolved.

Preparation of organic phase: A total of 80 mL of cyclohexane, 20 mL of trichoromethane, and 2 mL of span-80 were added to a 250 mL flask and were well-mixed in a beaker.

**Figure 1:**
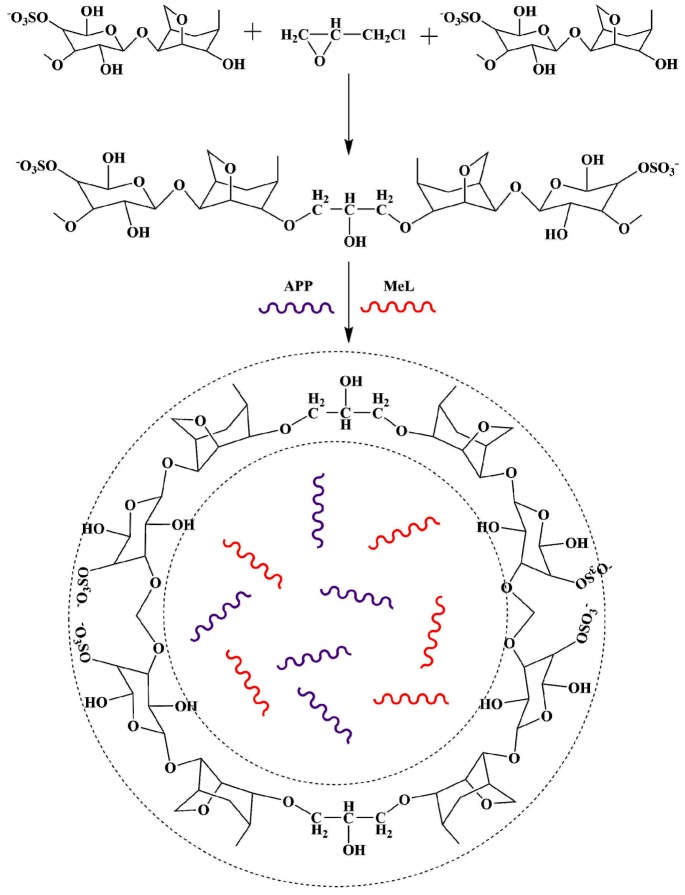
Preparation of the KC-IFR sample.

Preparation of KC-IFR: Suspension emulsion was prepared by first slowly adding a monomer aqueous solution to an organic phase. Then, the emulsion was emulsified for 10 min with the high-shear emulsifier to obtain a stable inverse suspension. This suspension emulsion was transferred into 250 mL three-necked flask provided with a stirrer. Then a solution of epichlorohydrin was added to the flask, and the reaction temperature was kept at 25 °C for 4 h. After the reaction completion, the suspension was washed thrice with anhydrous ethanol, and the supernatant was removed. Finally, it was dried under vacuum to obtain KC-IFR. The reaction process of KC-IFR is shown in Figure 1.

#### 2.2.2. Synthesis of BFG

The BFG was synthesized according to the literature [21]. A total of 960 mg of p-aminobenzoic acid, 280 mg of sodium hydroxide, 526 mg of sodium nitrite, and 6 mL of hydrochloric acid were dissolved in 80 mL of water and stirred for 45 min in an ice bath to prepare a diazonium salt solution. A total of 300 mg of graphite oxide was dissolved in an aqueous solution of 1% sodium dodecyl benzene sulfonate for 1 h, and then diazonium salt solution was added to the graphite oxide dispersion. The mixed solution was stirred in an ice water bath for about 4 h and stirring was continued at room temperature for 4 h. Finally, the supernatant was washed 3 times with ethanol and lyophilized to obtain BFG.

#### 2.2.3. Preparation of Flame-Retardant Coating

Flame-retardant EP coatings were prepared by different mass ratios of KC-IFR and BFG. The formulation is listed in Table 1. The preparation process was as follows: KC-IFR and BFG were firstly blended with waterborne epoxy resin using a pearl mill for 30 min. The curing agent and deionized water were added into the compound and stirred at room temperature for 30 min. The coating was scraped onto one side of a 100 × 100 × 1 mm^3^ steel plate, and the sample was cured at room temperature for one week before the test.

#### 2.2.4. Characterization

Fourier transform infrared spectroscopy (FTIR) was recorded on a Nicolet MNGNA-IR560 (Artisan Technology Group, Austin, TX, USA) with transition mode and the wave-number range between 400 and 4000 cm^−1^. Scanning electron microscope (SEM) was carried out on SEM JEOL JSM-6360LV, Japan equipped with energy dispersive X-ray spectroscopy (EDS). X-ray diffraction pattern (XRD) of the BFG was recorded on a D8 Advance X-ray diffractometer (Bruker, Karlsruhe, Germany) with Cu Kα radiation (λ = 0.154) and the scanning speed at 5°/min. Limited oxygen index (LOI) data were obtained using an oxygen index instrument (JF-3) (Jiangning Analysis Instrument Company, Nanjing, China) according to GB/T 2406-2009 standard. The dimensions of the specimens were 126 × 6.5 × 3 mm^3^. The vertical burning test was carried out on a CZF-3-type instrument (Jiangning Analysis Instrument Company, Nanjing, China) according to ASTM D3801-2010 standard. The dimensions of the specimens were 130 × 13 × 3 mm^3^. The back-temperature insulation test fixed the steel plate coated with the flame-retardant coating 1 cm above the flame with the coating facing down to ensure that the flame can burn the center of the coating. An infrared thermometer was fixed on the sample to ensure that the infrared temperature was measured at the center of the back of the steel plate. The back surface temperature of the steel plate was separated by 5 min, and the recording time was 60 min. Cone calorimeter tests were carried out on a Fire Testing Technology (FTT, England, UK) cone calorimeter. The specimens were irradiated at a heat flux of 50 kW/m^2^ according to ISO 5660-1 standard procedures. The dimensions of the specimens were 100 ×100 × 1 mm^3^. Thermogravimetric analyses (TGA) was tested on a STA 449C thermal analyzer (Selb, Germany) from 40 to 800 °C at a heating rate of 10 °C /min under nitrogen atmosphere.

## 3. Results and Discussion

### 3.1. Characterization of KC-IFR

**Figure 2:**
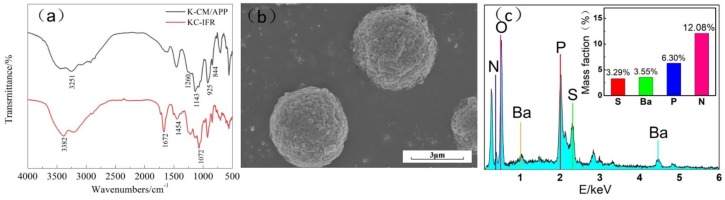
(**a**) Fourier transform infrared spectroscopy (FTIR) spectra of K-CM/APP and KC-IFR; (**b**) scanning electron microscopy (SEM) of KC-IFR; (**c**) energy dispersive X-ray spectroscopy (EDS) of KC-IFR.

The FTIR spectra of K-CM/APP and KC-IFR were shown in Figure 2a. In the FTIR spectrum of the K-CM/APP, the absorption peak of 1260 cm^−1^ was due to the symmetrical stretching vibration of O–S–O, the absorption peaks of 844 cm^−1^ and 925 cm^−1^ were attributed to the telescopic vibration of C–O–C, indicating the presence of KC. Moreover, the typical absorption peaks of APP include 3251 cm^−1^ (N–H), 1143 cm^-1^ (O–P–O), and 1260 cm^−1^ (P=O). In addition, the FT-IR spectrum of the KC-IFR had new characteristic absorption peaks. The absorption peak of 3382 cm^−1^ was due to the anti-symmetric stretching vibration of NH_2_, bending vibration absorption peak of NH at 1672 cm^−1^; the stretching vibration absorption peak of C=N at 1454 cm^−1^ indicated the presence of melamine. These results indicated the presence of KC, MEL, and APP in the KC-IFR.

The structure and morphology of the KC-IFR particle were shown in Figure 2b. It is clear that the surface of the KC-IFR particle was not smooth, and the surface had severe wrinkles and bulges. This is due to the melamine having poor water solubility, which affected the balling process of KC. However, the microspheres were uniformly dispersed and sized. This shows that the microspheres were well dispersed.

The elemental composition of the KC-IFR is shown in Figure 2c and shows the coexistence of P and N elements in KC-IFR particles. However, the content of N elements far exceeds that of P elements. This shows that the N element was not only the root in APP, but also a large part of the root in MEL. These results also suggested that successful preparation of the flame-retardant microsphere with KC coated APP and MEL.

### 3.2. Characterization of BFG

**Figure 3:**
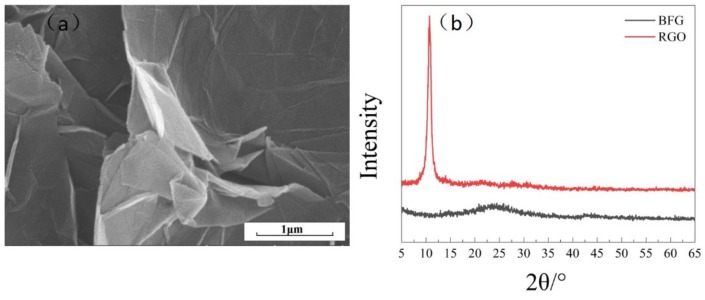
(**a**) SEM micrographs of BFG; (**b**) XRD pattern of BFG.

The SEM micrographs of BFG are shown in Figure 3a. It is clear that the BFG laminated structure was separated and there is no pile up and agglomeration between each other. This is due to p-aminobenzoic acid being a compound with many polar groups. This indicated that BFG has good dispersion and can improve the compatibility with polymer matrix. On the other hand, the XRD pattern of BFG and GO is shown in Figure 3b. According to the Bragg equation, the crystal plane spacing of GO was calculated to be 0.34 nm in the XRD pattern. This is due to graphite being oxidized by a strong oxidant, the surface was led into a large number of oxygen-containing functional groups, with the crystal lattice destroyed and the interplanar spacing becoming larger. In the XRD pattern of the BFG, the characteristic diffraction peak appears at 2θ = 23°, the diffraction peaks are markedly broadened and the peak was shifted to higher values of 2θ. This is due to the introduction of benzoic acid functional groups, resulting in the destruction of the integrity of RGO crystal structure, and most of the lamellae of BFG are stacked in disorder. In addition, the spacing between crystal planes was calculated to be 0.38 nm based on the Bragg equation. The crystal plane spacing was increased by 0.04 nm compared to that of GO. This indicates that the benzoic acid functional groups on the surface of BFG increase the interlayer spacing. In conclusion, the BFG synthesis was successful [21].

### 3.3. Intrinsic Fire Behavior of Flame-Retardant Coating

In order to evaluate the flame retardancy of the waterborne epoxy, the prepared waterborne epoxy and its composites were tested by LOI and UL-94 with the relevant data listed in Table 2. Pure epoxy EP0 cannot reach any rating in the UL-94 vertical burning test; the combustion was accompanied by a large number of droplets, and LOI value was only 19.7%. When the KC-IFR was added, the LOI value of the flame-retardant coating increased. Moreover, the V0 ratio was achieved in the UL-94 test without the appearance of melt drop. Almost the same results were observed in the LOI and UL-94 test for EP1 and EP2 samples, which indicated that KC-IFR played a major role in the flame-retardant system. Due to GO being easy to agglomerate and its poor dispersion, it does not function well to block oxygen. Therefore, the increase of LOI was not obvious. When BFG was added into the EP instead of GO, the amount of BFG added increased, the LOI values of EP3, EP4, and EP5 were also gradually increased. This is because of the improved dispersion of BFG within the matrix, which can form a barrier layer and effectively isolate the external oxygen.

**Figure 4:**
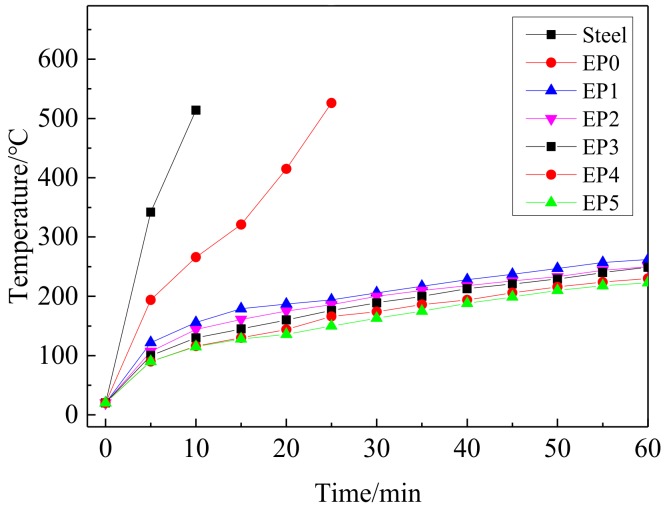
Back-temperature of EP curves for flame-retardant coatings.

Figure 4 shows the back-temperature insulation test curves for flame-retardant coating. As can be seen, the initial temperature of each coating was the same, the temperature of the pure steel sheet reached 500 °C within 10 min, and EP0 also reached 500 °C within 25 min. The heating rate of EP0 was slower in the early stage and the heating rate was faster in the late stage. Because the pure epoxy resin coating burned, the combustion absorbed a large amount of heat, and a thin layer of molten heat insulation was formed on the surface of the steel sheet. As the combustion continued, the molten heat insulation layer was rapidly oxidized. The rate of temperature rise of the steel sheet was also rapidly increased. The temperature rise of other flame-retardant coatings added with KC-IFR was obviously slowed down, because the APP was heated to dehydrate and decarburize the KC wall layer, and the MEL released gas to rapidly expand the char layer from the inside to form an expanded char layer, blocking the transfer of heat to the steel structure substrate [22]. In EP2, GO can participate in the char formation process of the coating, increasing the strength and density of the char layer, prolonging the oxidation time of the char layer. The addition of BFG to EP3, EP4, and EP5 not only allows BFG to be better dispersed in the coating, but also reduced the agglomeration of GO and enhanced the thermal barrier properties of the coating, better increasing the density of the coating. The EP5 fire-retardant coating incorporated 2% BFG with a higher amount of char residue and the best thermal barrier. This showed that the amount of char residue in the coating had an important influence on the flame-retardant insulation [23].

**Figure 5:**
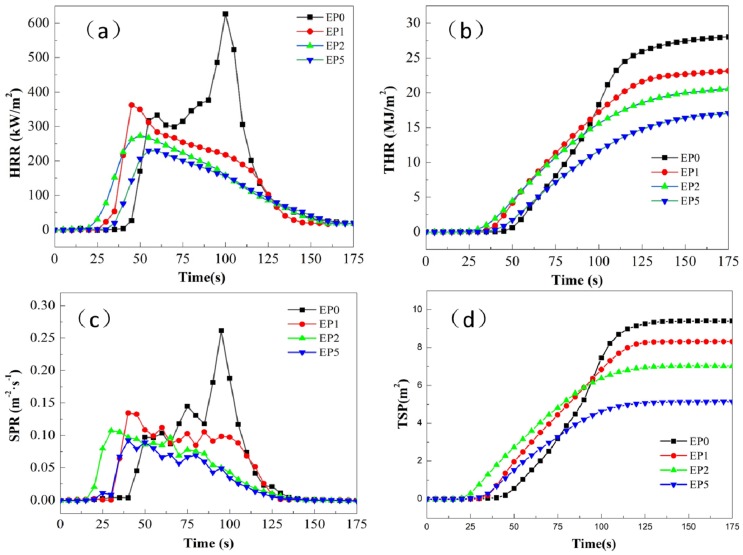
(**a**) HRR curves of flame-retardant coatings; (**b**) THR curves of flame-retardant coatings; (**c**) SPR curves of flame-retardant coatings; (**d**) TSP curves of flame-retardant coatings.

In order to further study the effects of the addition of KC-IFR and BFG on EP fire hazards, we performed cone calorimetry on pure EP and its composites containing KC-IFR and BFG. The heat release rate (HRR) in the cone calorimetry was one of the most significant parameters for evaluating the flame-retardant properties of materials. It can be seen from Figure 5 and Table 3 that the EP0 heat release rate peak (pHRR) was 627 kW/m^2^, and the pHRR of EP1, EP2, and EP5 were reduced by 42.3%, 56.5%, and 63.5%, respectively. The HRR curves of EP0 had two narrow and sharp heat release peaks, indicating that a large amount of heat was released in a short time, which increased the risk of violent combustion of the material. The HRR curves of EP1, EP2, and EP5 only have a wider peak, indicating that the heat was not released in a short time. This is because the KC-IFR was thermally expanded and formed a large amount of char layer with the coverage of the surface of the material, which can effectively prevent further combustion and reduce the risk of material banging [24]. Compared with the total heat release (THR) of EP0 (28 MJ/m^2^), the THR of EP1, EP2, and EP5 was significantly reduced. In particular, the THR of EP2 and EP5 were reduced to 21 MJ/m^2^ and 17 MJ/m^2^, respectively. It showed that GO had a further promoting effect on the char formation of the flame-retardant coating. Besides, the smoke release rate (SPR) of EP1, EP2, and EP5 were reduced by 50.0%, 57.7%, and 65.4% compared with EP0 (0.26 m^-2^·s^-1^). The total smoke production (TSP) of EP0 was 9.41 m^2^, while the TSP of EP1, EP2, and EP5 were 11.2%, 25.4%, and 45.5% lower than EP0. It is evidenced that the TSP values of EP5 were much lower than that of EP2. This is because BFG reduced the agglomeration of GO and increased its dispersion in the coating, thus providing better adsorption and barrier properties for the volatiles from combustion. Therefore, the excellent synergistic flame-retardant and smoke suppressing properties can be obtained with the presence of BFG and KC-IFR [25].

### 3.4. Analysis of Micromorphology

**Figure 6:**
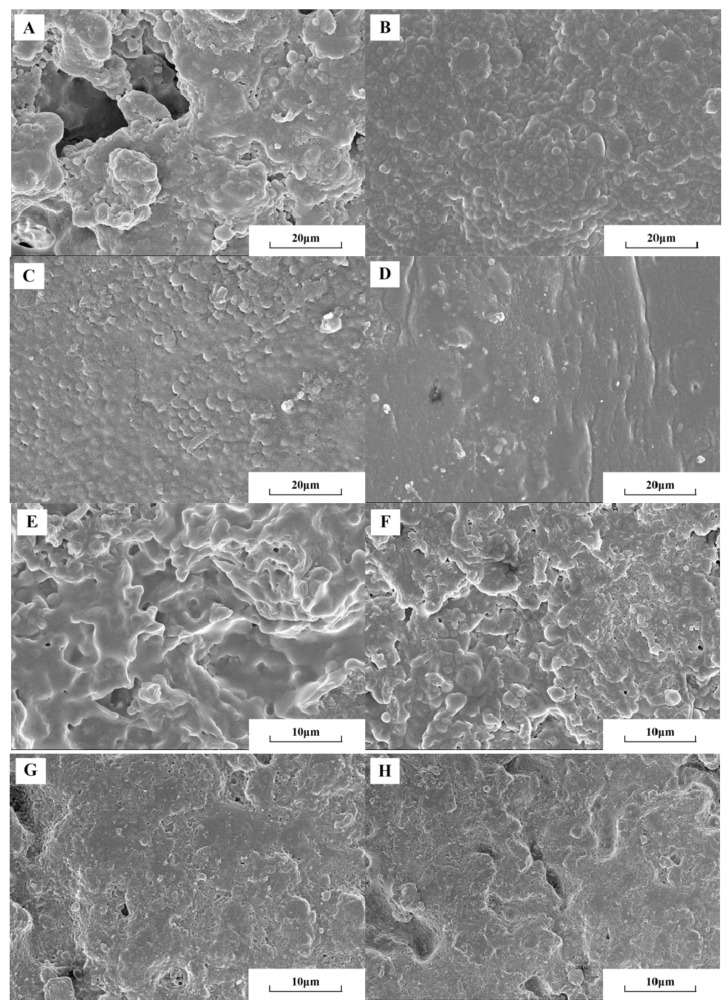
Scanning electron microscopy (SEM) micrographs of the surface and section of the flame-retardant coating. (**A**), (**B**), (**C**), and (**D**) were SEM of the surface of EP0, EP1, EP2, and EP5; (**D**), (**F**), (**E**), and (**G**) were SEM of the section of EP0, EP1, EP2, and EP5.

Figure 6 showed the SEM images of the surface and section of the flame-retardant coatings of EP0, EP1, EP2, and EP5 to illustrate the dispersion of the flame-retardant filler in the EP. As can be seen from the SEM images, the surface of EP0 was not flat, which was distributed with a large number of microporous defects. Its internal structure was also irregular with channel defects. There were micro-pleats on the surface and inside of the EP1. The coating was continuous, and no channel defects and aggregation occurred. The surface of EP2 was smooth and flat, continuously without defects. But there were still a small number of protrusions and wrinkles. However, the inside section was smooth with a few tiny holes. The addition of GO can effectively improve the curing process of the coating and enhance the integrity of the EP. The surface of EP5 was even more flat with no obvious wrinkles produced. Correspondingly, the internal structure was complete without obvious defects, which indicated that BFG had better effect on improving the integrity of EP [15].

**Figure 7:**
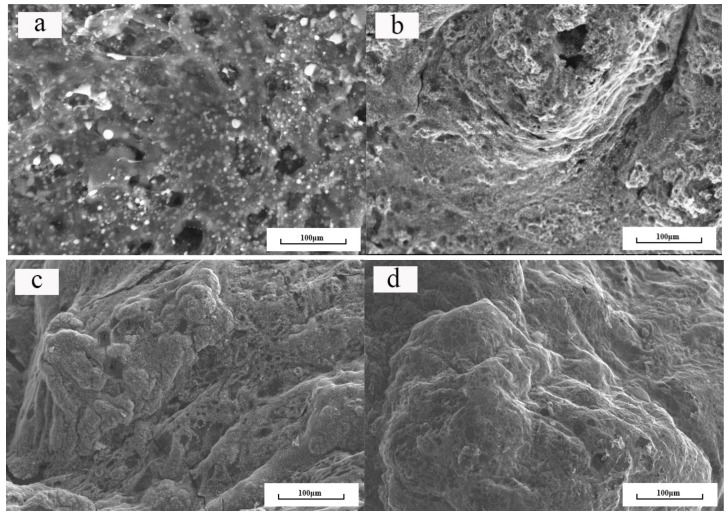
Scanning electron microscopy (SEM) micrographs of chars after the LOI test. (**a**) EP0; (**b**) EP1; (**c**) EP2; (**d**) EP5.

**Figure 8:**
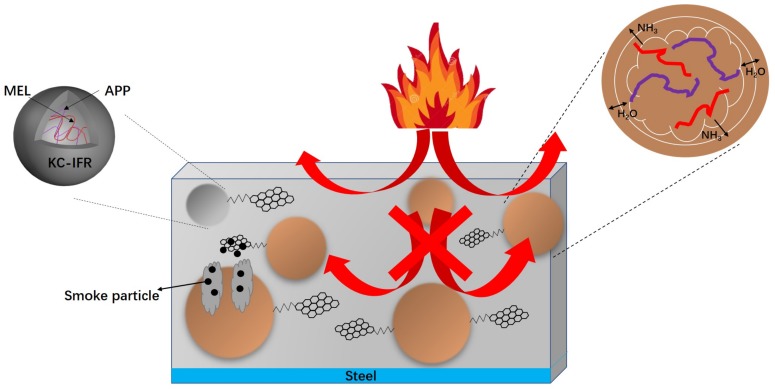
Schematic illustration of the flame-retardant mechanism of the flame-retardant coating.

The char residue can truly reflect the carbonization behavior of the polymer combustion process, which was beneficial to the analysis of the flame-retardant mechanism of the polymer. The structure and morphology of the char residue from the LOI test was analyzed by SEM as shown in Figure 7. As can be seen from the figure, the surface of EP0 exhibited a vitreous char layer, which was caused by the droplets produced by pure EP during combustion. The surface of the EP1 was covered by a large amount of surface roughening the char layer with the presence of small holes. This is because APP decomposed, which catalyzed the KC shell into char of KC-IFR. Besides, MEL generated a large amount of gas by heat, thereby forming a carbon layer with holes. The surface of the char layer of EP2 was slightly smoother, but it still had a large number of irregular char layers and holes. This is because GO was doped in the char layer during combustion, which can effectively improve the integrity and compactness of the char layer, thereby increasing the thermal barrier of the EP. Due to the easy agglomeration of GO individually, the flame retardancy of the EP was limited. However, with the modified BFG, EP5 formed a char layer with a smooth surface and high density and this char layer structure completely stopped the external heat source from the substrate, effectively preventing the heat from spreading. This is because BFG can be uniformly dispersed in the EP, which greatly improved the flame retardancy of the coating [26,27]. Upon the basis of the fire behavior, the flame-retardant mechanism is proposed, as illustrated in Figure 8.

### 3.5. Thermal Stability of Flame-Retardant Coating

**Figure 9:**
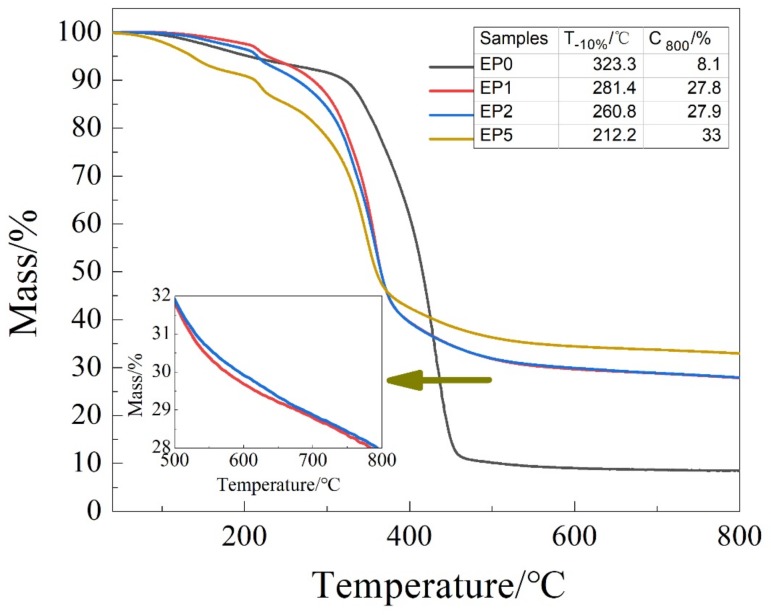
Thermogravimetric analysis curves of flame-retardant coating under N_2_.

Thermal stability and char forming ability of the flame-retardant coating were investigated by thermogravimetric analysis. Figure 8 gave the TGA profiles of the flame-retardant coating. The relative thermal stability of the samples was evaluated by the initial decomposition temperature (T_-10%_) and the char residual percentage at 800 °C (C_800_), as listed in Figure 9 [28].

It can be seen from the figure that T_-10%_ of the flame-retardant coating was lower than that of EP0. When the temperature reached 450 °C, EP1, EP2, EP5 exhibited better high temperature stability than EP0. The T_-10%_ of EP1 decreased due to the addition of KC-IFR, but the C_800_ increased from 8.1% to 27.8%. This is because the decomposition of APP in KC-IFR produced metaphosphoric acid and polyphosphoric acid catalytic to produce a stable char layer structure, and the protective substrate from continuous heat. When part of the KC-IFR was replaced by GO and BFG, respectively, the C_800_ of EP2 and EP5 reached 27.9% and 33%, respectively, indicating that GO and BFG can participate in the char formation of EP. Then, BFG can be well dispersed in EP and the EP5 finally promoted the formation of char structures, being able to withstand high temperature, thereby forming a barrier layer and preventing the transfer of external heat to the substrate [29].

## 4. Conclusions

In this study, a novel bio-based flame-retardant microsphere (KC-IFR) was prepared by using KC as the wall material, APP and MEL as the core material. Benzoic acid functionalized graphene (BFG) was also prepared. Then, KC-IFR and BFG were added into the EP as a flame-retardant system to prepare a flame-retardant coating. SEM tests showed that KC-IFR and BFG can be evenly distributed in EP. Various fire tests including LOI, UL-94, and cone calorimeter test demonstrated that the EP5 can achieve the best intrinsic fire-retardant and smoke suppression property with high LOI value (29.5%), UL-94 V-1 rating, and significant decrease in pHRR and TSP. The analysis of char residue showed that KC-IFR played a major role in flame-retardant coating. BFG can participate in the combustion of flame-retardant coating into charcoal to form dense char with better heat and oxygen resistance.

## Figures and Tables

**Table 1:** Formulation of coatings.

Sample	*m* (EP)/g	*m* (Curing Agent)/g	*m* (H_2_O)/g	*m* (KC-IFR)/g	*M* (GO)/g	*m* (BFG)/g
EP0	60.0	20.0	20.0	-	-	-
EP1	45.0	15.0	20.0	20.0	-	-
EP2	45.0	15.0	20.0	19.0	1.0	-
EP3	45.0	15.0	20.0	19.0	-	1.0
EP4	45.0	15.0	20.0	18.5	-	1.5
EP5	45.0	15.0	20.0	18.0	-	2.0

Note: “m” means mass; “-” means no such data.

**Table 2:** LOI and UL-94 date of coatings.

Sample	EP0	EP1	EP2	EP3	EP4	EP5
LOI/%	19.7	28.7	28.9	29.1	29.5	29.8
UL-94	No rating	V-0	V-0	V-0	V-0	V-0
Droplet	Yes	No	No	No	No	No

**Table 3:** Cone calorimetry test data of the coating.

Sample	pHRR (kW/m^2^)	THR (MJ/m^2^)	pSPR (m^−2^·s^−1^)	TSP (m^2^)
EP0	627	28	0.26	9.41
EP1	362	23	0.13	8.36
EP2	273	21	0.11	7.02
EP5	229	17	0.09	5.13
